# Malignant Triton Tumors in Sisters with Clinical Neurofibromatosis Type 1

**DOI:** 10.1155/2015/405351

**Published:** 2015-05-31

**Authors:** Basnet Alina, Jofre A. Sebastian, Capo Gerardo

**Affiliations:** ^1^Trinitas Regional Medical Center, Seton Hall University of Health and Medical Sciences, Elizabeth, NJ 67202, USA; ^2^Department of Internal Medicine, Trinitas Regional Medical Center, Elizabeth, NJ 67202, USA; ^3^Touro College of Osteopathic Medicine, 230 W 125th Street No. 1, New York, NY 10027, USA; ^4^Department of Hematology and Oncology, Trinitas Comprehensive Cancer Center, Elizabeth, NJ 07202, USA

## Abstract

Malignant triton tumors (MTTs) are rare and aggressive sarcomas categorized as a subgroup of malignant peripheral nerve sheath tumors (MPNSTs). MTTs arise from Schwann cells of peripheral nerves or existing neurofibromas and have elements of rhabdomyoblastic differentiation. We report the occurrence of MTTs in two sisters. The first patient is a 36-year-old female who presented with left sided chest wall swelling. She also had clinical features consistent with neurofibromatosis type 1 (NF-1). Debulking of the mass showed high-grade malignant peripheral nerve sheath tumor with skeletal muscle differentiation (MTT). The patient was treated with ifosfamide and adriamycin along with radiation. Four years after treatment, she still has no evidence of disease recurrence. Her sister subsequently presented to us at the age of 42 with left sided lateral chest wall pain. Imaging showed a multicompartmental retroperitoneal cystic mass with left psoas involvement. The tumor was resected and, similarly to her sister, it showed high-grade malignant peripheral nerve sheath tumor with rhabdomyoblastic differentiation (MTT). The patient was started on chemotherapy and radiation as described above.

## 1. Introduction

Malignant triton tumors (MTTs) are a histological subgroup of the malignant peripheral nerve sheath tumors (MPNSTs) with focal component of heterologous skeletal muscle differentiation [1]. MPNSTs are of neuroectodermal origin and arise from the Schwann cells of peripheral nerves or nearby cells, usually through independent perineural differentiation or within preexisting neurofibromas [1–4]. MTTs derive their name from the triton salamander which possesses the ability to develop supernumerary limbs composed of mixed neurogenic and muscular components following sciatic nerve implantation on its dorsal surface [5, 6].

MTTs account for 5–10% of MPNSTs [1, 3] and occur, on average, during the 3rd to 5th decade [1]. Previous studies have reported a 50–70% occurrence of these tumors in the setting of neurofibromatosis type 1 (NF-1) [4]. The tumor behaves very aggressively, with a high rate of local recurrence peaking at 43–50% [1, 7]. The five-year survival rate is reported to be 14% [3] as compared to 34–60% for MPNSTs alone [1]. The median time to progression is 6 months [3]. MTTs pose many diagnostic challenges for pathologists because of the nuanced findings histologically, often resulting in a misdiagnosis to the conventional MPNST [1, 3]. The correct diagnosis relies heavily on histologic criteria, and immunohistochemistry becomes crucial for narrowing the differential diagnosis [8]. MTTs behave more aggressively; these patients have a shorter metastases-free interval and shorter survival as compared to MPNSTs [4, 7]. Thus, MTTs must be differentiated from conventional MPNSTs in order to ensure prompt and aggressive treatment [1, 3].

Once diagnosed, physicians face the difficulty of determining the proper therapeutic regimen. Due to the rarity of this type of tumor; there is no treatment consensus available yet. Wide surgical excision or debulking of the tumor with negative margins and successive radiotherapy is currently often employed [4]. The role of chemotherapy, albeit unproven in efficacy, continues to be used in many cases and it must be noted that this reflects a distinct difference from MPNSTs, where adjuvant chemotherapy is sometimes not used after debulking due to the lack of survival benefit seen as mentioned by Stucky et al. [1, 9, 10]. Moreover, some investigators have employed different combinations of chemotherapy in higher doses in order to achieve a better result in MTT patients [7].

Here we discuss two unique cases describing two sisters who were diagnosed with MTTs only a few years apart. Their presentation stands out from the literature available in their interpatient relationship, gender, and age of presentation. Although MTT is described as an exceedingly rare tumor, the fact that it occurred in two siblings in conjunction with clinical NF-1 is of interest. To our knowledge, no prior cases of MTTs occurring in siblings have been described in the literature. Additionally, MTTs in the setting of NF-1 have been described in a patient population of primarily young males [11]. Our patients are, however, female and slightly older than the age associated with NF-1. Finally, despite the aggressive behavior that characterizes this tumor type and the poor prognosis of this disease, even with rigorous therapeutic regimens, it is worthy of noting that one of the sisters remains disease-free even after 4 years of treatment.

## 2. Case Report

**Figure 1:**
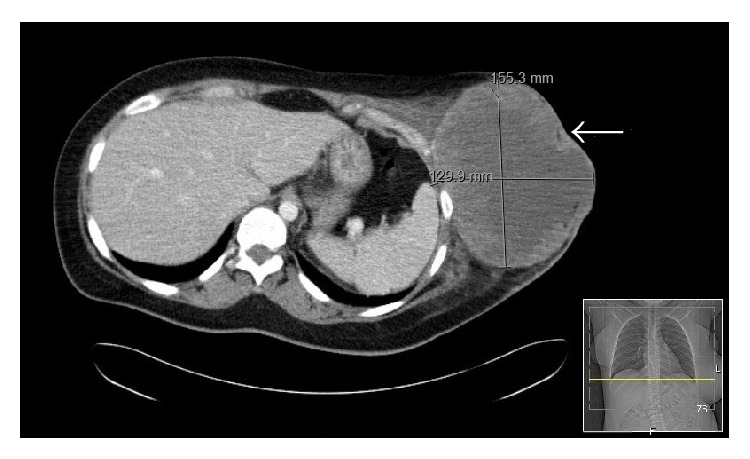
Arrow pointing to the soft tissue mass in the left lateral chest wall with its measurement.

**Figure 2:**
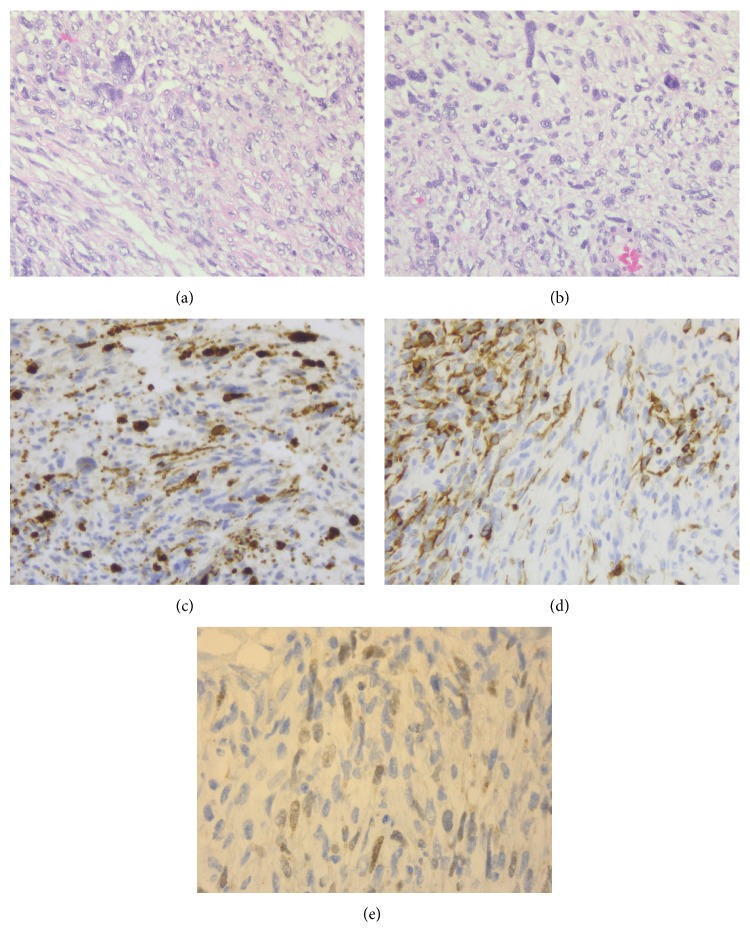
(a) and (b) Malignant triton tumor (MTT) cells. Tumor cellularity and spindle shaped pleomorphic cells are apparent. Interspersed eosinophilic muscle fibers are within the field (H&E). (c) Positive immunohistochemical stain with desmin highlighting presence of striated muscle within tumor. (d) Positive immunohistochemical stain with S-100. This verifies the presence of neural component within the tumor. (e) Positive immunohistochemical stain with MyoD transcription factor demonstrating myogenic component of tumor.

We describe the presentation of malignant triton tumors in two siblings who are familiar to our cancer center. The first patient is a 36-year-old female with past medical history of asthma who presented to us with complaints of left sided chest wall swelling and mild pain that progressed over a few days. She denied the history of smoking, alcohol, or drugs. Family history was significant for colon cancer in father and maternal aunt. She was not sexually active. On examination, she was found to have axillary freckling, café au lait spots, and mild cognitive impairment. The findings were consistent with NF-1. There was no associated hepatosplenomegaly noted on examination. On imaging, a very large soft tissue mass measuring 15 cm by 15 cm by 13 cm in the left lower anterior lateral chest wall with possible ulceration and axillary lymphadenopathy was seen. The mass infiltrated the adjacent chest wall musculature; however, the ribs and lungs were commented as being intact and clear, respectively (Figure 1). At the time, it was thought to be a possible soft tissue abscess. Thereafter, debridement of the left sided chest mass was undertaken. Due to the rarity of the histologic findings, our pathology department referred this case to Memorial Sloan Kettering for a second opinion. Their findings are quoted as “mainly monotonous undifferentiated spindle cells arranged in intersecting fascicles and vague storiform pattern with high mitotic activity.” Immunohistochemical (IHC) stains revealed tumor cells positive for desmin, vimentin, and myogenin as well as focal reactivity for S-100 (Figure 2). On the other hand, markers CD31, CK, EMA, and CD57 were reported to be negative. These results were, thus, consistent with the diagnosis of a high-grade malignant peripheral nerve sheath tumor with focal divergent skeletal muscle differentiation (triton tumor) in the background of fat and fibrous tissue. After discussion in the multidisciplinary round, we decided to give her adjuvant chemotherapy consisting of ifosfamide and adriamycin for four cycles along with pre- and postchemotherapy radiation. She was started on combination chemotherapy of ifosfamide 2 g/m^2^ with mesna 2 g/m^2^ alternating with adriamycin 75 g/m^2^ every other week for first two cycles four weeks apart and then continued with ifosfamide and mesna for the rest of the two cycles without adriamycin. Concomitant radiation was given after the 2nd cycle and continued till the 3rd chemotherapy cycle. The radiation therapy was applied to the left abdominal wall, with a total dose of 65.4 Gy, with initial photon fields, followed by shrinking fields with electrons with 33 dose fractions during the course of two months. She currently follows up in our cancer center and has no evidence of disease recurrence four years after her treatment.

**Figure 3:**
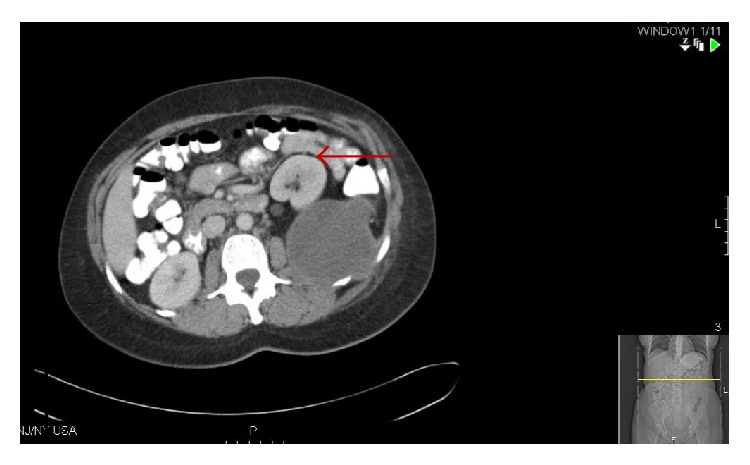
Red arrow pointing to the displaced left kidney by the soft tissue mass.

**Figure 4:**
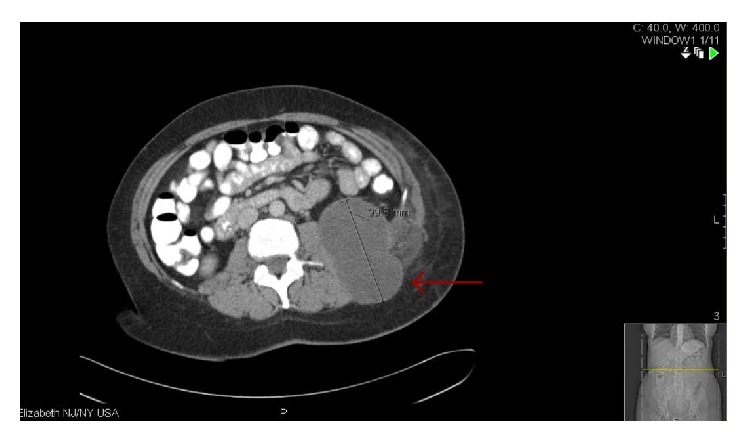
Red arrow pointing to the cystic component of the soft tissue mass with its measurement.

**Figure 5:**
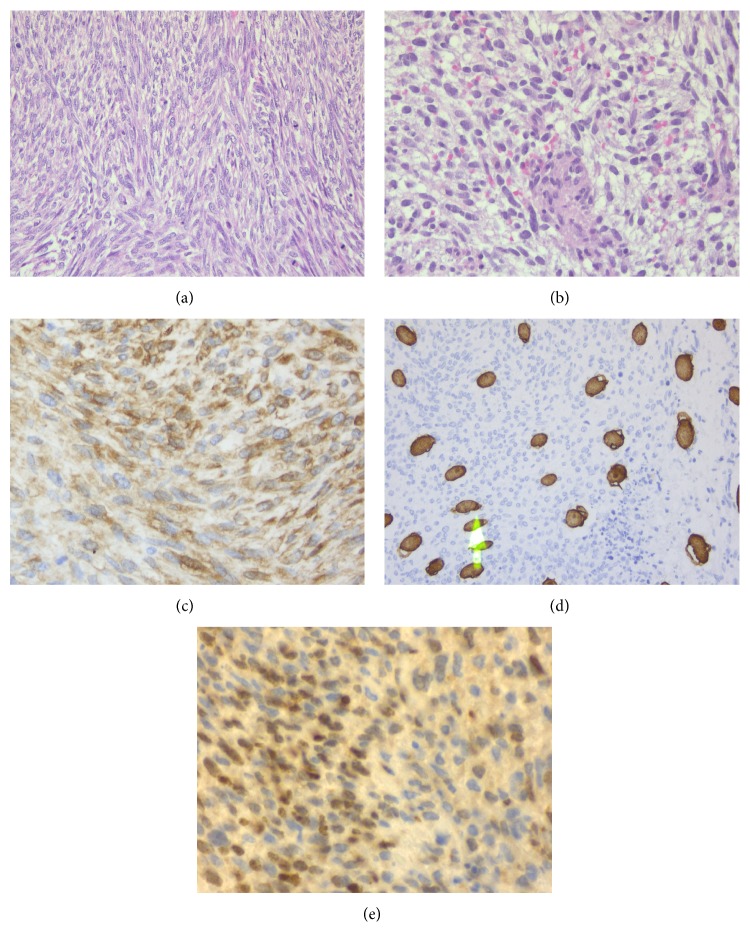
(a) Malignant triton tumor (MTT) cells. Spindle, fasciculated, and pleomorphic malignant schwannoma cells are appreciable with numerous mitotic figures in the field. (b) Eosinophilic rhabdomyoblasts within malignant schwannoma cells. (c) Positive immunohistochemical stain with NF-200 highlighting neurofilamentous component of tumor. (d) Positive immunohistochemical stain with desmin. (e) Positive immunohistochemical stain with MyoD transcription factor demonstrating myogenic component of tumor.

Unfortunately, her younger sister presented to us a few years later at the age of 42 with complaints of pain in the left lateral chest wall with radiation to the left flank that was worsening over 3-4 months. There was no trauma preceding the event. She had no past medical history and was a nonsmoker and nonalcoholic. She was not sexually active. On examination, café au lait spots were also noted on her along with mild cognitive impairment. There was fullness on the left side of the chest underneath the left ribs with some tenderness to palpation but no axillary lymphadenopathy or splenomegaly. On imaging, a multicompartmental retroperitoneal cystic mass in the posterior pararenal space was observed. It extended through the posterior abdominal wall, measuring 10 cm by 7.7 cm by 10.4 cm, and exerted a compressive effect on the left psoas muscle while simultaneously displacing the left kidney anteriorly. Bulky mediastinal and paratracheal lymphadenopathy was additionally observed (Figures 3 and 4). There was no intrathoracic or pleural involvement. She underwent debulking surgery for the mass. Subsequently, histopathological examination of the retroperitoneal mass showed high-grade malignant peripheral nerve sheath tumor with rhabdomyoblastic differentiation (triton tumor) (Figure 5). The tumor was described to have spindle and pleomorphic malignant schwannoma cells, scattered rhabdomyoblasts, and focal areas of necrosis. Immunohistochemical stains are positive with NF-200, desmin, and MyoD. The Ki67 stain demonstrated a proliferative rate of 90%, which was also evident by the abundancy of mitotic figures. She is currently undergoing chemotherapy with ifosfamide with adriamycin at the exact same dosing schedule received by her sister. We plan to continue this treatment regimen for four cycles with concurrent radiotherapy.

## 3. Discussion

MPNSTs with elements of rhabdomyoblastic differentiation are termed as malignant triton tumors (MTTs). MTTs are referred to as a mosaic tumor due to their muscular and neurogenic components [12]. Masson was the first to report the presence of rhabdomyoblasts in this neurogenic tumor and described them as rhabdomyomas of the nerve [13]. It was not until 1973 that Woodruff et al. introduced the term MTT [14].

The incidence of MPNSTs is 1 per 100,000 cases [3]. MTTs account for 5–10% of MPNSTs [1]. NF-1 (von Recklinghausen's disease) has been associated with an increased incidence of MPNSTs and MTTs [2]. MTTs are associated with NF-1 disease in 50–70% of the cases, occurring mainly in young males. The remaining cases are comprised of sporadic growths, occurring mostly in older females. A postradiotherapy manifestation has also been described [6]. Unlike the cases described in the literature, our patients are older female patients with MTTs in association with NF-1.

The most established theory regarding the histopathogenesis of MTTs is that multipotent neural crest cells of ectomesenchymal origin are capable of divergent differentiation to both nerve and muscle components [3]. It has also been proposed that Schwann cells in neurogenic tumor could be stimulated by motor nerves to differentiate into rhabdomyoblastic components. Alternatively, E. Kamperis et al. demonstrated in a rat model that neoplastic Schwann cells possess the capacity for mesenchymal differentiation into rhabdomyoblasts [6].

In the majority of reported cases, triton tumors are located across peripheral nerves, usually close to the spine, commonly in the head and neck region [3]. Few cases have previously been observed in the mediastinum and extremities [1–3]. Less common sites include buttocks and the retroperitoneum [3]. Cases as rare as intracardiac presentation of MTTs have been reported [15, 16]. MTTs typically present as a rapidly enlarging mass with associated pain and compressive symptoms to adjacent organs.

Woodruff et al. established the original histological criteria for the diagnosis of the malignant triton tumors. He stated that the tumors must arise along the course of a peripheral nerve or in a preexisting neurofibroma in patients with NF-1. Additionally, the tumors must display identical growth characteristics of Schwann cells. Finally, they must contain “bona fide” rhabdomyoblasts originating from the body of peripheral nerve tumor and not from extension or metastasis of an extrinsic rhabdomyosarcoma [14]. Daimaru et al. further broadened the definition to encompass the sporadic forms in patients without NF-1, which, he argued, are microscopically comparable malignant schwannomas with focal rhabdomyoblasts, and tumors consisting predominantly of rhabdomyoblastic differentiation with focal Schwann cell elements occurring within a nerve or in patients with NF-1 [17].

Today, the diagnosis of MTT is generally made according to these criteria as well as immunohistochemical findings for cell origin tracing. Nerve sheath differentiation is confirmed by S-100 protein and Leu-7 (CD57) positivity. Rhabdomyoblastic differentiation is confirmed by immunohistochemical positivity for desmin, actin, and myogenin [3, 12]. Through these findings, the diagnosis of MTT can be positively made and the proper course of treatment can be pursued.

However, one major area of concern for clinicians is the rate of tumor occurrence and regrowth after appropriate treatment. Local recurrence rate varies from 22% to 43% [1, 7, 18] and metastasis has been reported to occur in 20–50% of cases [7, 18]. Recurrence is dependent on several factors, including tumor location, extent of surgical excision, degree of differentiation and growth pattern, and performance status and comorbidities [6]. Tumors originating from head and neck have lower rates of recurrence, whereas tumors originating from the trunk, buttocks, retroperitoneum, and CNS have lower rates of recurrence. Wong et al. reported that MTTs involving nonextremity sites have a higher likelihood to recur.

Lymphatic invasion and lymph node involvement has not been reported in patients with MTT [6]. The most common site of metastasis is lung [1], but common extra pulmonary metastases include bone, liver, peritoneum, and the central nervous system. PET/CT is helpful in detecting distant metastases [1]. Anecdotal views vary regarding the impact of NF-1 status on survival. Zakzouk et al. report that NF-1 is associated with more incidence of metastasis [19], while Kamperis et al. mention, in a literature review, that NF-1 does not affect prognosis [6]. Few reports mention shorter survival in cases of MTT not associated with NF-1 [14].

Currently, there is no consensus regarding the treatment guidelines for malignant triton tumors. Several authors have proposed recommendations, including radical excision, radical excision followed by radiotherapy and chemotherapy, and excision with high dose radiotherapy. However, as for any other sarcoma, resection of the tumor with wide margins followed by radiotherapy is the recommended treatment. The need for chemotherapy has not been clearly defined [19]. Some recent reports suggest that neoadjuvant and adjuvant therapies can eradicate micrometastases [11]. In the case of suboptimal cytoreduction, a second more extensive surgical excision should always be pursued [6]. Proposed chemotherapeutic regimens include PEI (cisplatin, etoposide, and ifosfamide) as first-line chemotherapy and IA (ifosfamide and adriamycin) or MAID (mesna, doxorubicin, ifosfamide, and dacarbazine) as second-line treatment. There have been previously reported cases of MTTs that responded favorably to treatment with isotretinoin (a retinoid analogue) and interferon-*α* [6]. In conclusion, there are conflicting recommendations in the literature regarding the optimal treatment of MTTs [6, 9, 11, 19]. We can only agree that further investigations and case reports are necessary in order to continue contributing to our knowledge of MTTs, their clinical courses, and treatment outcomes.

In our case reports, we treated the first sister with four cycles of adjuvant ifosfamide and adriamycin along with radiotherapy after wide surgical excision and reresection of the tumor. She responded well to it and remains disease-free even after 4 years of initial diagnosis. We are treating the second sister with the same chemotherapy regimen along with concurrent radiotherapy. She is currently responding well to treatment with few side effects. We will continue to monitor her and observe her treatment response.

We present the cases of MTTs in two sisters with clinical neurofibromatosis. Our cases are unique due to their sisterly presentation within a decade of each other. This is in contrast to MPNSTs, which have been previously described in siblings. Additionally, MTTs in the setting of NF-1 have not been seen in our patient demographics: middle-aged females. Given the aggressive nature of this tumor subtype, one of the sisters has remained disease-free for four years after initiation of adjuvant chemotherapeutic therapy. We are hopeful of the treatment potential for her sister. Additionally, the information learned regarding this disease type may help shed light on the role of chemotherapy in care of patients diagnosed with MTTs.
